# Electrospun 3D Fibrous Scaffolds for Chronic Wound Repair

**DOI:** 10.3390/ma9040272

**Published:** 2016-04-06

**Authors:** Huizhi Chen, Yan Peng, Shucheng Wu, Lay Poh Tan

**Affiliations:** 1 School of Materials Science and Engineering, Nanyang Technological University, 50 Nanyang Avenue, Singapore 639798, Singapore; chen0881@e.ntu.edu.sg; 2 Interdisciplinary Graduate School, Nanyang Technological University, 50 Nanyang Avenue, Singapore 639798, Singapore; 3 School of Mechanical Engineering, Ngee Ann Polytechnic, 535 Clementi Road, Singapore 599489, Singapore; alicepengyan@gmail.com (Y.P.); wsh3@np.edu.sg (S.W.)

**Keywords:** electrospinning, tissue engineering, wound healing, PLGA, fibrous scaffolds, surface modification

## Abstract

Chronic wounds are difficult to heal spontaneously largely due to the corrupted extracellular matrix (ECM) where cell ingrowth is obstructed. Thus, the objective of this study was to develop a three-dimensional (3D) biodegradable scaffold mimicking native ECM to replace the missing or dysfunctional ECM, which may be an essential strategy for wound healing. The 3D fibrous scaffolds of poly(lactic acid-co-glycolic acid) (PLGA) were successfully fabricated by liquid-collecting electrospinning, with 5~20 µm interconnected pores. Surface modification with the native ECM component aims at providing biological recognition for cell growth. Human dermal fibroblasts (HDFs) successfully infiltrated into scaffolds at a depth of ~1400 µm after seven days of culturing, and showed significant progressive proliferation on scaffolds immobilized with collagen type I. *In vivo* models showed that chronic wounds treated with scaffolds had a faster healing rate. These results indicate that the 3D fibrous scaffolds may be a potential wound dressing for chronic wound repair.

## 1. Introduction

Chronic wounds represent one of the most significant unmet healthcare burdens in the world today and they are a main complication in diabetic patients. Chronic wounds are non-healing wounds that have failed to carry on a spatial and temporal continuum of events to achieve wound closure [1]. The goal of wound care would be to regenerate tissues by restoring structural and functional properties as before injury. Current therapies for chronic wounds include growth factors administration [2], but a limited amount of growth factors may be insufficient to restore tissue homeostasis. Although tissue-engineered skin substitutes have also been used for chronic wounds, there are several intrinsic shortcomings such as fragile epidermal grafts, creation of new wounds in autografts, and possible transmission of infectious diseases and immune rejection in the use of allografts or xenografts [3,4].

Deficiencies in the extracellular matrix (ECM) and the accumulation of devitalized tissues are significant characteristics of non-healing wounds [5]. The introduction of a biofunctional construct to replace the missing or dysfunctional ECM may be beneficial. Ideally, such replacement should closely mimick natural ECM, providing physical and chemical cues for recruiting nearby skin cells into the wound bed. From this aspect, the mode of three-dimensional (3D) fibrous polymer scaffolds modified with natural ECM protein is of interest.

Electrospinning is an easy and low-cost approach to fabricate fibrous matrices. A polymer solution is loaded in a capillary tube and confined by its surface tension, and the ejection of polymer solution is accomplished when it is exposed to an electric field and the electrostatic repulsion overcomes the surface tension. The solvent evaporates when the solution-jet travels in air, leaving behind polymeric fibers which will deposit on the collector [6,7,8]. Thus, continuous fibers are collected to form a non-woven fabric that mimics the fibrous network of native ECM, and the fiber sizes are closely similar to ECM fibrils [9,10]. It was reported that the electrospun fiber matrices offer morphologic cues that result in enhanced cell response. These fibrous scaffolds have presented improved cell attachment, proliferation, migration, and gene expression signature [11,12,13,14,15]. The improved cell performance from electrospun scaffolds may render accelerated tissue repair and they may thus work as attractive matrices for tissue engineering [16].

The basal limitation with conventional electrospinning is that the scaffold produced is usually two-dimensional (2D) dense mats rather than 3D porous structures, which impedes cell infiltration near the superficial surface. Several methods have been explored to increase the porosity of electrospun fibrous matrices to facilitate cell ingrowth. One common strategy involves easily eliminated materials (such as salt and water-soluble polymer) during electrospinning and selective removal after electrospinning [17,18,19,20]; however, the scaffold’s stability is often altered after sudden elimination. Another strategy is the application of ultrasonication where physical manipulation can enlarge the pore size of electrospun scaffolds [21], but it does not produce a true 3D scaffold, especially in terms of the thickness. In place of the traditional collector, cotton-ball-like scaffolds could be electrospun in a spherical collector with an array of needles [22]; however, this is difficult to replicate due to precise control over parameters such as the dimensions of the spherical dish, needle length, needle distance, *etc*.

In this study, we utilized the liquid collector [23], which is a simple and easy-to-reproduce 3D fibrous scaffold with high porosity, via electrospinning. Biodegradable synthetic polymer was used as the material and the surface was chemically modified with natural ECM protein to develop a biologically active matrix, providing both structural support and biological recognition for cell growth and recruitment which is a crucial part of the wound healing process. We hypothesize that these 3D fibrous scaffolds with physical and chemical clues will help to facilitate the repair of chronic wounds *in vivo*.

## 2. Results

### 2.1. Characterization of 3D Fibrous Scaffolds

**Figure 1:**
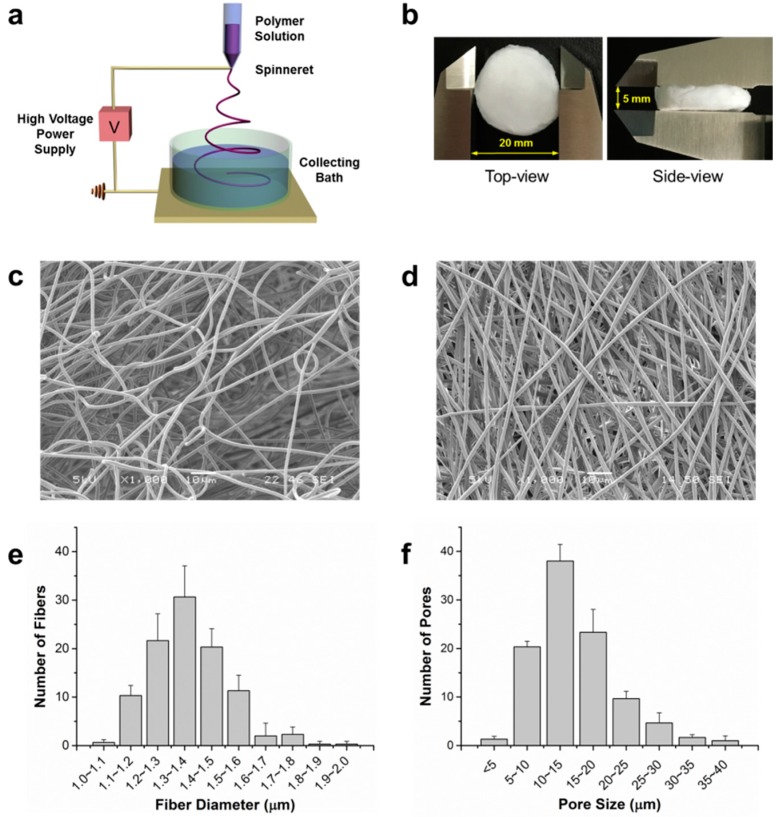
(**a**) Illustration of liquid-collecting electrospinning setups; (**b**) Relative shape and size of spongiform fibrous scaffolds; (**c**) Scanning electron microscopy (SEM) characterization of electrospun scaffolds collected from liquid bath and (**d**) conventional flat platform; (**e**) Distribution of fiber diameter and (**f**) pore size within 3D electrospun fibrous scaffolds. One hundred measurements of fiber diameter and pore size were randomly made from each scaffold; *n* = 3.

The relative shape and size of scaffolds produced from liquid-collecting electrospinning (Figure 1a) are shown in Figure 1b. The scaffolds were 3D spongiform and shaped into cylinders with a diameter of 20 mm and thickness of 5 mm. To check the fiber morphology and porous structure of both scaffolds fabricated by electrospinning with the liquid collector and conventional collector, scanning electron microscopy (SEM) was utilized. The 3D fibrous scaffolds (Figure 1c) collected by the liquid bath exhibited large pores in both horizontal and vertical directions, while interweaving fibers were closely packed vertically in the 2D non-woven mat (Figure 1d) fabricated by the conventional system. From SEM characterization images, the scaffold parameters were further examined quantitatively. The fiber diameter of the 3D fibrous scaffolds ranged from 1.0 to 1.8 µm (Figure 1e) with an average diameter of 1.34 ± 0.12 µm. The pore sizes ranged from 5 to 40 µm, and most of them were between 5 and 20 µm with an average pore size of 14.54 ± 6.47 µm (Figure 1f).

### 2.2. Surface Modification with Collagen Type I

The immobilization of proteins on a poly(lactic acid-co-glycolic acid) (PLGA) fiber surface was accomplished as described [24]. Briefly, carboxylic groups were generated through the hydrolysis of PLGA by immersion in NaOH solution, and chemical bonds were formed between the amine groups from the proteins and the carboxylic groups on the PLGA fiber’s surface. P-C50, P-C100 and P-C500 designated the scaffolds immobilized with collagen type I at concentrations of 50, 100 and 500 µg/mL, respectively, and the control scaffold without surface modification was designated as P-BLK. The immobilized protein was quantified by microBCA assay, and the results are shown in Figure S1. The protein amount of P-C100 was 10.27 ± 4.06 µg per scaffold, which was significantly larger than that of P-C50 (4.77 ± 0.70 µg per scaffold), and no significant difference was detected between P-C100 and P-C500 (12.19 ± 7.07 µg per scaffold).

**Figure 2:**
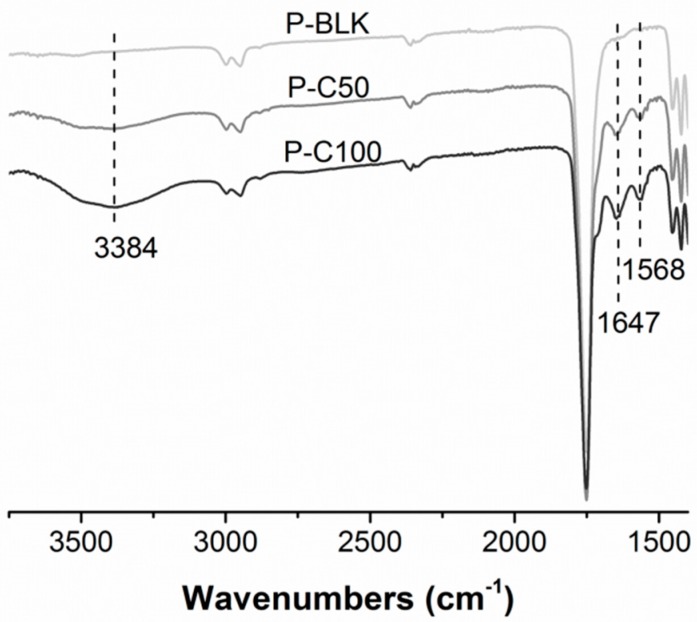
Attenuated total reflection Fourier transform infrared spectroscopy (ATR-FTIR) analysis of P-BLK (light gray), P-C50 (gray), and P-C100 (blank).

Attenuated total reflection Fourier transform infrared spectroscopy (ATR-FTIR) was employed to characterize the surface chemistry of various scaffolds, and the spectra are shown in Figure 2. The intense peaks at 1750 cm^−1^ found in common belong to the C=O stretching of PLGA backbone; however, three specific peaks were detected at 3384, 1647, and 1568 cm^−1^ in the P-C50 and P-C100 spectra. The extensive peak detected at 3384 cm^−1^ is relevant to N-H stretching from primary and secondary amines, while peaks at 1647 and 1568 cm^−1^ correspond to N-H bending from primary amines. These results indicated amine-containing substances were present in P-C50 and P-C100.

### 2.3. Cellular Infiltration and Proliferation

**Figure 3:**
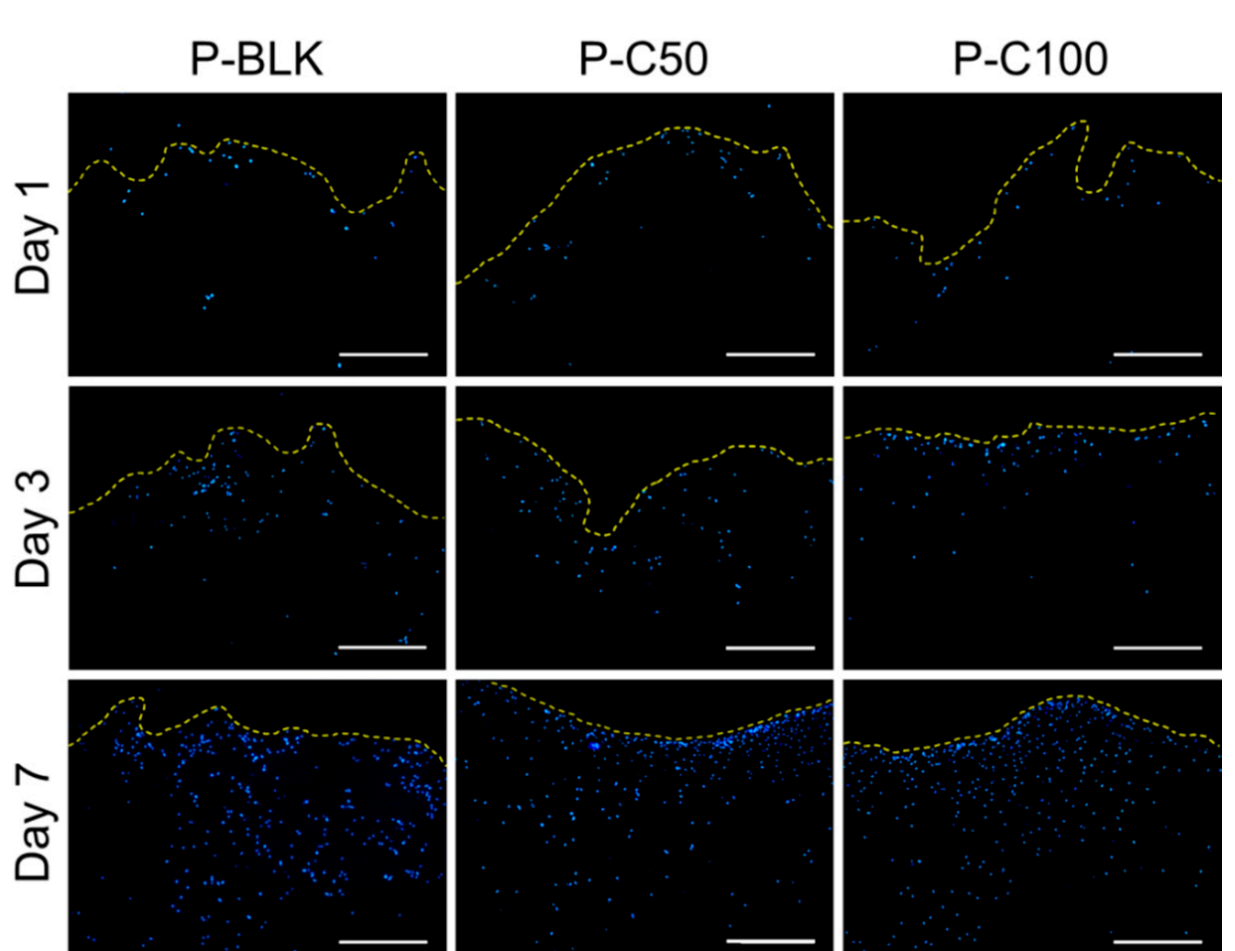
Fluorescence microscope images of 4’,6-diamidino-2-phenylindole (DAPI) stained cross-sections of P-BLK, P-C50, and P-C100 scaffolds seeded with human dermal fibroblasts (HDFs) on day 1, 3, and 7. The yellow dotted lines indicate the top margin of scaffolds. Section thickness = 30 µm. Scale bar = 500 µm.

The cellular infiltration into the scaffolds was examined by studying the cross-sections of the scaffolds using cryosectioning coupled with 4’,6-diamidino-2-phenylindole (DAPI) staining. Figure 3 shows cross-sectional images of various scaffolds seeded with human dermal fibroblasts (HDFs) on the top surface after one, three, and seven days. Over the culturing time, cells gradually proliferated and invaded the scaffolds. On day 1 the cells had adhered on the scaffolds and their infiltration was limited to the upper layer (~200 µm), while the cells penetrated deep into ~700 µm on day 3. By day 7, cells completely migrated into the inner part of the scaffolds at a depth of ~1400 µm. The cellular infiltration patterns were comparable on all 3D scaffolds in both the presence and absence of collagen type I.

**Figure 4:**
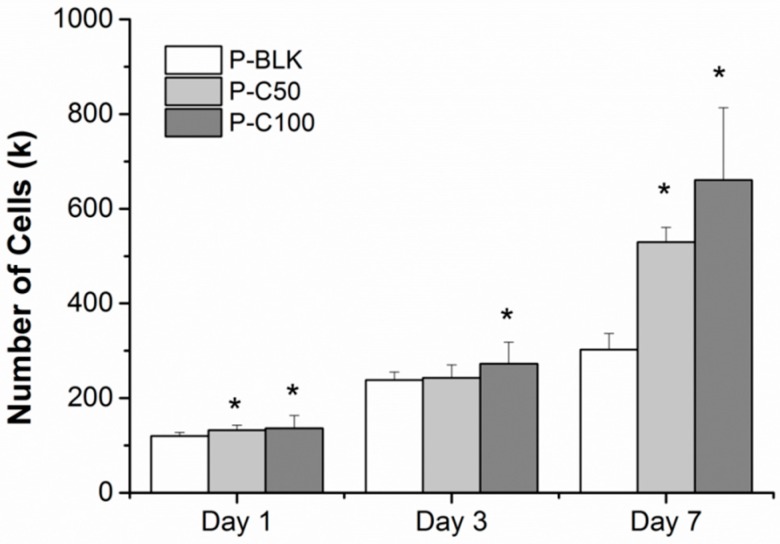
Human dermal fibroblasts (HDFs) proliferated on P-BLK (white), P-C50 (light gray), and P-C100 (gray) within seven days. * The cell number was significantly greater as compared with P-BLK (*p* < 0.05). Error bar represents standard deviation of means; *n* = 3.

The cellular proliferation on various scaffolds was quantitatively evaluated and is as shown in Figure 4. The cell loading density was 100 k per scaffold. After one day of culturing, the cell counts on P-C50 and P-C100 were 133.91 ± 11.61 k and 136.32 ± 27.13 k, respectively, while a significantly lower cell number (117.34 ± 11.04 k) was detected for P-BLK. On day 3, the cell number for all scaffolds increased to over 200 k. They were 238.38 ± 16.65, 242.82 ± 27.47, and 272.50 ± 45.66 k for P-BLK, P-C50, and P-C100, respectively, and a significant difference was only exhibited between P-BLK and P-C100. The most noticeable change was observed between day 3 and day 7. Over this time, the cell number on P-BLK increased to 302 ± 33.39 k, while the cell number on P-C50 and P-C100 markedly increased to 529.72 ± 31.16 k and 661.05 ± 152.73, respectively, which was significantly greater than that on P-BLK. No significant difference was shown between P-C50 and P-C100 over the culturing time.

### 2.4. In Vivo Wound Healing

**Figure 5:**
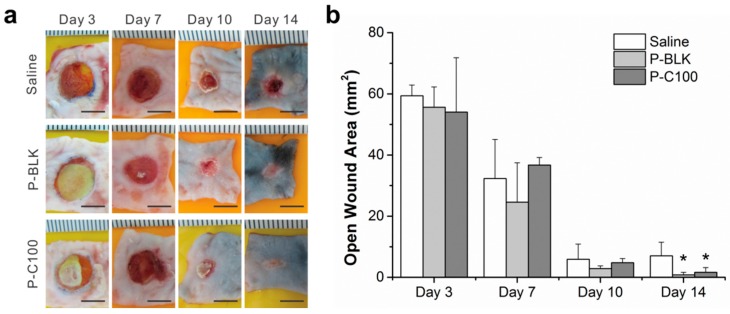
Macroscopic images (**a**) and wound closure measurement (**b**) of saline-, P-BLK-, and P-C100-treated wound biopsies taken at day 3, 7, 10, and 14 post-wounding. Scale bar = 5 mm. * On day 14, both the open wound areas of P-BLK and P-C100 treatments were smaller than that of saline (*p* < 0.05). Error bar represents standard deviation of means; *n* = 3.

Macroscopic observation and quantitative evaluation of wound closure on P-BLK, P-C100, and saline-treated diabetic mice were studied after three, seven, 10, and 14 days post-wounding, as shown in Figure 5. The wounds diminished with time for all groups, and there was no significant difference between groups from day 3 to day 10. However, wounds were nearly covered with P-BLK and P-C100 treatment on day 14, and the open wound areas were significantly smaller in comparison with that on the saline-treated mice.

**Figure 6:**
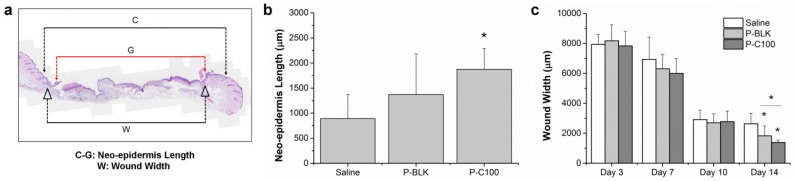
Schematic representation of the histologically stained wound section (**a**) and measurements of neo-epidermis length at day 3 (**b**) and wound width at indicated days (**c**). C is the distance between the first hair follicles on the wound edge. G is the distance between the advancing margins of neo-epidermis. Neo-epidermis length = C-G. W (wound width) is the distance between the cut ends of skin connective tissue. * At day 3, neo-epidermis length of P-C100 treating was larger than that of saline treatment (*p* < 0.05). * At day 14, both wound width of P-BLK and P-C100 treating were smaller than that of saline treatment (*p* < 0.05). Error bar represents standard deviation of means; *n* = 3.

Histomorphometric analysis of wound sections was investigated by hematoxylin and eosin staining (Figure S2), and quantitative measurements of the neo-epidermis length and wound width were made from the stained wound sections (Figure 6a). Neo-epidermis formations were observed at the initial wound healing phase (day 3), and the neo-epidermis length of the P-C100 treatment was significantly greater than that of the saline treatment (Figure 6b). By day 10, the epithelial gap of all groups diminished to zero, representing a completely re-epithelialized wound. The epithelial gap is defined as the distance between the advancing margins of the neo-epidermis, which is indicated with red arrows in Figure S2. Wound width, the distance between the end cuts of the skin connective tissue, decreased with time for all groups (Figure S2), and quantitative assessment showed that wound closure was significantly greater in P-BLK and P-C100 treatments after 14 days (Figure 6c). However, no significant difference between groups was detected from day 3 to day 10.

## 3. Discussion

The ECM-mimicking microenvironment of electrospun scaffolds could enhance cellular behaviors in various aspects [25,26,27,28]. However, cellular growth and infiltration are usually limited to the superficial layer of the flat, sheet-like fiber mat prepared by traditional electrospinning, presenting a significant obstacle in developing tissue replacement using this method [22]. From that point of view, we developed a 3D network scaffold incorporating fibrous morphologies and interconnected pore structures by altering the collector. In traditional electrospinning, a flat, grounded platform is used as the collector, and fibers are deposited on the platform layer by layer to form a densely-packed 2D structure (Figure 1d). Instead of collecting in air, collecting in liquid can decrease the bulk density of the fiber matrix [23]. In order to achieve fiber penetration and sinking into the liquid rather than floating on the liquid surface, the surface tension and density should be lower as compared to the polymer solution. Thus, IPA and surfactant Kollphor^®^ P188 were added to reduce the density and surface tension.

Fiber formation was influenced by the solution and processing parameters during electrospinning, among which viscosity and polarity of the polymer solution are vital to spinnability and fiber morphology. Solution viscosity depends on the inherent viscosity and concentration of the polymer. In this study, using PLGA with the inherent viscosity of 1.05 dL/g, the polymeric solution at the concentration of 15% produced continuous and smooth fibers. PLGA dissolved in the sole solvent of TFE resulted in coiled fibers. The addition of chloroform with a lower dielectric constant produced coil-free fibers, and hence we hypothesized that a stronger electric field leads to coiled fibers. The reality that the application of higher voltage also produced coiled fibers has verified the hypothesis. Beyond the intrinsic properties of the solution, the processing parameters including voltage, flow rate, and tip-to-target distance were optimized to obtain defect-free fibers. Taken together, 3D fibrous scaffolds with interconnected 5~20 µm pores were successfully developed and fabricated by liquid-collecting electrospinning.

PLGA as a biodegradable, biocompatible and FDA-approved polyester has been considered an attractive candidate for scaffolding material in regenerative medicine [29,30]; however, its bio-inert surface is incapable of supporting cellular adhesion. Cells will attach on the PLGA surface only after protein deposition on the PLGA surface, from the culture medium or as secreted by cells [31]. Collagen type I is the major component of skin-native ECM with abundant arginine-glycine-aspartate (RGD) peptide to stimulate cell attachment [32]. It is widely accepted that the incorporation of native protein is desirable as a promising scaffolding material to modulate cellular behaviors through the cell-substrate interactions [33,34,35,36]. With the purpose of activating cell-material interactions, we engineered the PLGA fiber surface with collagen type I through chemical modification and we qualitatively examined it using ATR-FTIR. The successful grafting of collagen type I was indicated by the distinct presence of amine signals (3384, 1647, and 1568 cm^−1^) on the spectra of P-C50 and P-C100. Saturation of grafting at 100 µg/mL collagen was manifested by the amounts of immobilized protein, which were comparable between P-C100 and P-C500. The efficacy of collagen-immobilized PLGA for improved cellular proliferation has been exhibited in the fact that P-C50 and P-C100 supported more cells after one day of culturing (initial period). Over seven days of culturing, the significantly greater cell number for P-C50 and P-C100 clearly demonstrated that PLGA scaffolds modified with collagen type I tend to promote cell proliferation. No obvious improvement was detected between P-C50 and P-C100, which may indicate that improved cellular proliferation was fulfilled by 4.77 ± 0.70 µg collagen per scaffold.

Engineered scaffolds with interconnected pore structures allow cells and nutrients to penetrate into the internal environment, building a 3D ECM-mimicking architecture, while the optimal pore size is dependent on specific tissue. It was reported that HDFs have a propensity for pore sizes of 6 to 20 µm, where larger pores (>20 µm) lead to cells growing along fibers instead of branching out in a 3D configuration [37]. As an *in vitro* cell infiltration study, HDFs were seeded on the surface of a 3D fibrous scaffold and were shown to migrate into the inner part of all scaffolds gradually over time, reaching as deep as ~1400 µm after seven days of culturing. Therefore, the fibrous scaffolds with interconnected 5~20 µm pores seem to be attractive for skin cell ingrowth. The cellular infiltration patterns were similar on all 3D scaffolds with or without immobilized collagen type I, indicating that the highly porous structure appears to have a greater impact on cell infiltration than on the surface properties for this scaffold-material combination.

Although *in vitro* studies are important due to the accessibilities and controlled experimental conditions, it is really difficult to mimic the *in vivo* response. The *in vivo* wound healing experiments were processed on diabetic mice. Measurements of the open wound area and wound width showed that the wound closure of the diabetic mice was significantly accelerated by the 3D fibrous scaffolds in both the absence and presence of collagen within 14 days post-wounding, and scaffolds with collagen seemed to promote neo-epidermis formation in the initial wound healing phase. It is difficult to extrapolate the results of animal studies to the case of actual human exposure due to the difference in skin tension and all animal experiments being conducted in a sterile environment; however, animal studies are good starting points for new technologies. Fiber scaffolds have been widely investigated as a skin substitute based upon their remarkable architecture, as they possess similar mechanical properties as normal human skin, protect the wound area from bacterial infection and support cell respiration and proliferation [8,29,38,39]. Thus, 3D fibrous scaffolds seemed to be beneficial for chronic wounds, providing mechanical support and guidance for skin cell growth and then facilitating skin regeneration. Collagen as the predominant ECM of skin tissue plays a significant role in wound healing, increasing the intensity and integrity [40], and allowing the secretion of collagen type I from HDFs to be triggered when cultured on porous PLGA scaffolds [41]. This may be why P-C100 did not show a significant difference in wound healing as compared to P-BLK.

## 4. Materials and Methods

### 4.1. Materials

PLGA with molar ratio of 50/50 and inherent viscosity of 1.05 dL/g was purchased from Purac (Gorinchem, Netherlands). Tetrafluoroethylene (TFE), Kolliphor^®^ P188, 4-morpholineethanesulfonic acid (MES), EDC, and NHS were purchased from Sigma Aldrich (St. Louis, MO, USA). Chloroform was purchased from Fisher Scientific (Waltham, MA, USA). Collagen type I was purchased from Merck Millipore (Darmstadt, Germany). Fluoroshield mounting medium with DAPI was purchased from Abcam (Cambridge, UK). HDFs were purchased from Life Technologies (Fisher Scientific, Waltham, MA, USA). High glucose Dulbecco’s Modified Eagle Medium (DMEM) and PBS were purchased from Lonza (Basel, Switzerland). Fetal bovine serum (FBS) and antibiotic-antimycotic were purchased from Gibco (Fisher Scientific, Waltham, MA, USA).

### 4.2. Scaffolds Fabrication and Characterization

PLGA granules were dissolved at 15% in a solvent mixture of 7:3 (v:v) TFE and chloroform with stirring to form clear, homogeneous, and viscous solution. The PLGA solution was loaded into a plastic syringe and electrospun at flow rate of 0.5 mL/h through a 25 gauge blunt needle with the electrospinning apparatus NANON-01A (MECC, Fukuoka, Japan). A bath containing 7:3 (v:v) isopropyl alcohol and distilled deionized (DI) water with 0.05% Koppiphor^®^ P188 was used as collector. The bath was placed 4 cm vertically from the needle tip, and a voltage of 18 kV was applied. The electrospinning setups were illustrated as Figure 1a. The eventual dimension of scaffolds can be controlled by customized molds. For SEM characterization, the scaffolds were freeze-dried to retain the scaffolds structure. To quantify fiber diameter and pore size, measurements were made from 100 fibers and pores taken randomly in SEM images.

Surface modification was carried out as previously [24] with minute modification. Briefly, the scaffolds were immersed in 0.05% NaOH solution at room temperature for 30 min. Upon completion of hydrolysis, the samples were rinsed with DI water thoroughly. The hydrolyzed scaffolds were incubated in a mixture of 40 mM EDC and 80 mM NHS with 50 mM MES buffer (pH = 6) for 4 h at 4 °C. Activated scaffolds were washed with DI water thoroughly and soaked in collagen type I solution at concentration of 50, 100, and 500 µg/mL overnight at 4 °C. The samples were then washed with phosphate buffered saline (PBS).

The morphology of the scaffolds was examined with SEM at the working voltage of 5 kV. All samples were sputter-coated with a filmy layer of gold prior to SEM observation. Fiber diameter and void pore size were analyzed from SEM images with ImageJ software. The chemistry propertied of scaffolds was analyzed by ATR-FTIR. To quantify the immobilized protein, scaffolds were dissolved by chloroform and PBS was added immediately to extract the protein, which was quantified by microBCA assay.

### 4.3. Cellular Infiltration and Proliferation

HDFs were cultured in DMEM containing 10% FBS and 1% antibiotic/antimycotic mixture and the culture medium was replenished every two to three days. The cells were always incubated at 37 °C with humidified atmosphere of 5% CO_2_. Prior to cell seeding, scaffolds were sterilized by UV irradiation at 254 nm for 60 min. To study cell infiltration and proliferation, the adherent HDFs were detached by 0.05% trypsin and a cell suspension of 1 × 10^5^ cells was loaded on scaffolds top surface of 400 mm^2^. Cellular response was examined by collecting the scaffolds after one, three, and seven days. For each time point at least three specimens were examined for each group.

At the specified time points, scaffolds were collected and fixed with 4% paraformaldehyde to analyze cellular infiltration. After washing with PBS, they were soaked into 15% sucrose and then transferred into 30% sucrose. The scaffolds were then embedded in OCT freezing media overnight for efficient penetration and frozen at −20 °C. Cryosections of 30 µm thickness were used for analysis, and immunofluorescence staining was carried out by mounting with DAPI. Images were visualized by a fluorescence microscope and captured with a 4× objective lens.

To measure cellular proliferation, cell counting kit-8 (CCK-8; Dojindo Molecular Technologies, Rockville, MD, USA) was used. At the appropriate time point, scaffolds loaded with cells incubated in a mixture of 1:10 (v:v) CCK-8 reagent and cell culture medium at 37 °C for 2 h prior to detect absorbance at 450 nm. The cell number was calculated by absorbance standards of known cell numbers.

### 4.4. In Vivo Wound Healing

All animal experiments were approved and carried out in obedience to the directives of the Institutional Animal Care and Use Committee of Nanyang Technological University. The mice had free access to water and standard mouse chow for acclimatization period of one week. Thereafter, the food was replaced with high fats diet containing 58% fats and water was replaced with 30% fructose water. Monitoring of mice weight and food intake was implemented throughout the study. The high fed diet and high sucrose (HF-HS) induced mice would be obese and diabetic after four to six weeks of diabetogenic diet and blood glucose level of these mice will be measured (approx. 473 ± 14.6 mg/dL) before start of experiments.

A mixture of ketamine (80 mg/kg) and xylazine (10 mg/kg) was injected intraperitoneally to anesthetize mice prior to surgery. Circular full-thickness excisional splint wounds of 10 mm were created as previously described [42]. After surgery, various 3D scaffolds were applied topically to the wounds and covered by an occlusive dressing. Mice were euthanized by CO_2_ inhalation at day 3, 7, 10, and 14 post-wounding, wounds including small margin of surrounding skin were removed. Images of wounds were captured using a digital camera, and a ruler was appended in each image to allow measurement calibration. Surface wound area was quantified using Adobe Photoshop CS6 software. For histological analysis, wound biopsies were fixed in 4% paraformaldehyde and embedded in OCT freezing medium. Cryosections of 8 µm thickness were stained according to routine hematoxylin and eosin protocol. For each time point at least three wounds were examined for each group.

### 4.5. Statistical Analysis

Mean and standard deviation (SD) were measured for each group of experimental data, and the results presented are representative data sets. Significant difference among the experimental groups was determined by *t*-test using OriginPro software and two-tailed hypothesis. *p* values that were less than 0.05 were considered to be statistically significant.

## 5. Conclusions

Electrospun 3D scaffolds were developed to provide a stereoscopic structure of fibers with interconnected pores ranging from 5 to 20 µm. The fibrous morphology possessed a remarkable similarity to native ECM, and the pore structure facilitated HDFs infiltration at a depth of 1400 µm after seven days culturing. Surface modification with collagen type I promoted cellular proliferation. The treatment of fibrous scaffolds accelerated chronic wound closure within 14 days. These evaluations clearly demonstrate that the presence of 3D fibrous scaffolds can modulate the cellular behaviors and regeneration rate of skin tissue.

## Supplementary Materials

The following are available online at www.mdpi.com/1996-1944/9/4/272/s1.

## References

[1] Falanga V. (2005). Wound healing and its impairment in the diabetic foot. Lancet.

[2] Embil J.M., Papp K., Sibbald G., Tousignant J., Smiell J.M., Wong B., Lau C.Y., Canadian Becaplermin Study Group (2000). Recombinant human platelet-derived growth factor-bb (becaplermin) for healing chronic lower extremity diabetic ulcers: An open-label clinical evaluation of efficacy. Wound Repair Regen..

[3] Greaves N.S., Iqbal S.A., Baguneid M., Bayat A. (2013). The role of skin substitutes in the management of chronic cutaneous wounds. Wound Repair Regen..

[4] Widgerow A.D. (2014). Bioengineered skin substitute considerations in the diabetic foot ulcer. Ann. Plast. Surg..

[5] Blakytny R., Jude E.B. (2009). Altered molecular mechanisms of diabetic foot ulcers. Int. J. Low. Extremity Wounds.

[6] Li D., Xia Y.N. (2004). Electrospinning of nanofibers: Reinventing the wheel?. Adv. Mater..

[7] Sill T.J., von Recum H.A. (2008). Electrospinning: Applications in drug delivery and tissue engineering. Biomaterials.

[8] Zhang Y.Z., Lim C.T., Ramakrishna S., Huang Z.M. (2005). Recent development of polymer nanofibers for biomedical and biotechnological applications. J. Mater. Sci. Mater. Med..

[9] Barnes C.P., Sell S.A., Boland E.D., Simpson D.G., Bowlin G.L. (2007). Nanofiber technology: Designing the next generation of tissue engineering scaffolds. Adv. Drug Deliv. Rev..

[10] Smith L.A., Ma P.X. (2004). Nano-fibrous scaffolds for tissue engineering. Colloids Surf. B Biointerfaces.

[11] Kim C.H., Khil M.S., Kim H.Y., Lee H.U., Jahng K.Y. (2006). An improved hydrophilicity via electrospinning for enhanced cell attachment and proliferation. J. Biomed. Mater. Res. Part B Appl. Biomater..

[12] Kuppan P., Vasanthan K.S., Sundaramurthi D., Krishnan U.M., Sethuraman S. (2011). Development of poly(3-hydroxybutyrate-co-3-hydroxyvalerate) fibers for skin tissue engineering: Effects of topography, mechanical, and chemical stimuli. Biomacromolecules.

[13] Lee I.S., Kwon O.H., Meng W., Kang I.K. (2004). Nanofabrication of microbial polyester by electrospinning promotes cell attachment. Macromol. Res..

[14] Pan H., Jiang H.L., Chen W.L. (2006). Interaction of dermal fibroblasts with electrospun composite polymer scaffolds prepared from dextran and poly lactide-co-glycolide. Biomaterials.

[15] Patel S., Kurpinski K., Quigley R., Gao H.F., Hsiao B.S., Poo M.M., Li S. (2007). Bioactive nanofibers: Synergistic effects of nanotopography and chemical signaling on cell guidance. Nano Lett..

[16] Kumbar S.G., James R., Nukavarapu S.P., Laurencin C.T. (2008). Electrospun nanofiber scaffolds: Engineering soft tissues. Biomed. Mater..

[17] Baker B.M., Gee A.O., Metter R.B., Nathan A.S., Marklein R.A., Burdick J.A., Mauck R.L. (2008). The potential to improve cell infiltration in composite fiber-aligned electrospun scaffolds by the selective removal of sacrificial fibers. Biomaterials.

[18] Nam J., Huang Y., Agarwal S., Lannutti J. (2007). Improved cellular infiltration in electrospun fiber via engineered porosity. Tissue Eng..

[19] Guimaraes A., Martins A., Pinho E.D., Faria S., Reis R.L., Neves N.M. (2010). Solving cell infiltration limitations of electrospun nanofiber meshes for tissue engineering applications. Nanomedicine.

[20] Ekaputra A.K., Prestwich G.D., Cool S.M., Hutmacher D.W. (2008). Combining electrospun scaffolds with electrosprayed hydrogels leads to three-dimensional cellularization of hybrid constructs. Biomacromolecules.

[21] Lee J.B., Jeong S.I., Bae M.S., Yang D.H., Heo D.N., Kim C.H., Alsberg E., Kwon I.K. (2011). Highly porous electrospun nanofibers enhanced by ultrasonication for improved cellular infiltration. Tissue Eng. Part A.

[22] Blakeney B.A., Tambralli A., Anderson J.M., Andukuri A., Lim D.J., Dean D.R., Jun H.W. (2011). Cell infiltration and growth in a low density, uncompressed three-dimensional electrospun nanofibrous scaffold. Biomaterials.

[23] Yokoyama Y., Hattori S., Yoshikawa C., Yasuda Y., Koyama H., Takato T., Kobayashi H. (2009). Novel wet electrospinning system for fabrication of spongiform nanofiber 3-dimensional fabric. Mater. Lett..

[24] Wen F., Chang S., Toh Y.C., Teoh S.H., Yu H. (2007). Development of poly (lactic-co-glycolic acid)-collagen scaffolds for tissue engineering. Mater. Sci. Eng. C.

[25] Pham Q.P., Sharma U., Mikos A.G. (2006). Electrospinning of polymeric nanofibers for tissue engineering applications: A review. Tissue Eng..

[26] Li H., Wen F., Chen H., Pal M., Lai Y., Zhao A.Z., Tan L.P. (2016). Micropatterning extracellular matrix proteins on electrospun fibrous substrate promote human mesenchymal stem cell differentiation toward neurogenic lineage. ACS Appl. Mater. Interfaces.

[27] Wong H.K., Lam C.R.I., Wen F., Chong S.K.M., Tan N.S., Chan J., Pal M., Tan L.P. (2016). Novel method to improve vascularization of tissue engineered constructs with biodegradable fibers. Biofabrication.

[28] Li H.Q., Wong Y.S., Wen F., Ng K.W., Ng G.K.L., Venkatraman S.S., Boey F.Y.C., Tan L.P. (2013). Human mesenchymal stem-cell behaviour on direct laser micropatterned electrospun scaffolds with hierarchical structures. Macromol. Biosci..

[29] Kumbar S.G., Nukavarapu S.P., James R., Nair L.S., Laurencin C.T. (2008). Electrospun poly(lactic acid-co-glycolic acid) scaffolds for skin tissue engineering. Biomaterials.

[30] Makadia H.K., Siegel S.J. (2011). Poly lactic-co-glycolic acid (plga) as biodegradable controlled drug delivery carrier. Polymers.

[31] Ma Z.W., Mao Z.W., Gao C.Y. (2007). Surface modification and property analysis of biomedical polymers used for tissue engineering. Colloids Surf. B Biointerfaces.

[32] Uitto J., Olsen D.R., Fazio M.J. (1989). Extracellular matrix of the skin: 50 years of progress. J. Investig. Dermatol..

[33] Wang S., Taraballi F., Tan L.P., Ng K.W. (2012). Human keratin hydrogels support fibroblast attachment and proliferation *in vitro*. Cell Tissue Res..

[34] Tambe N., Di J., Zhang Z., Bernacki S., El-Shafei A., King M.W. (2015). Novel genipin-collagen immobilization of polylactic acid (pla) fibers for use as tissue engineering scaffolds. J. Biomed. Mater. Res. Part B Appl. Biomater..

[35] Zhu Y.B., Gao C.Y., Liu X.Y., Shen J.C. (2002). Surface modification of polycaprolactone membrane via aminolysis and biomacromolecule immobilization for promoting cytocompatibility of human endothelial cells. Biomacromolecules.

[36] Elbert D.L., Hubbell J.A. (1996). Surface treatments of polymers for biocompatibility. Annu. Rev. Mater. Sci..

[37] Lowery J.L., Datta N., Rutledge G.C. (2010). Effect of fiber diameter, pore size and seeding method on growth of human dermal fibroblasts in electrospun poly(epsilon-caprolactone) fibrous mats. Biomaterials.

[38] Bhattarai S.R., Bhattarai N., Yi H.K., Hwang P.H., Cha D.I., Kim H.Y. (2004). Novel biodegradable electrospun membrane: Scaffold for tissue engineering. Biomaterials.

[39] Lai H.J., Kuan C.H., Wu H.C., Tsai J.C., Chen T.M., Hsieh D.J., Wang T.W. (2014). Tailored design of electrospun composite nanofibers with staged release of multiple angiogenic growth factors for chronic wound healing. Acta Biomater..

[40] Panchatcharam M., Miriyala S., Gayathri V.S., Suguna L. (2006). Curcumin improves wound healing by modulating collagen and decreasing reactive oxygen species. Mol. Cell. Biochem..

[41] Yang W.S., Roh H.W., Lee W.K., Ryu G.H. (2006). Evaluation of functions and tissue compatibility of poly (d,l-lactic-co-glycolic acid) seeded with human dermal fibroblasts. J. Biomater. Sci. Polym. Ed..

[42] Wang X.S., Ge J.F., Tredget E.E., Wu Y.J. (2013). The mouse excisional wound splinting model, including applications for stem cell transplantation. Nat. Protocols.

