# Supplementary material for: Electrospun 3D Fibrous Scaffolds for Chronic Wound Repair
Source: Materials (Basel). 2016 Apr 6;9(4):272. doi: 10.3390/ma9040272 (PMC5502965; doi:10.3390/ma9040272)
Supplement: Supplementary file 1 [file materials-09-00272-s001.pdf]

Supplementary Materials: Electrospun 3D Fibrous Scaffolds for Chronic Wound Repair

Huizhi Chen, Yan Peng, Shucheng Wu and Lay Poh Tan

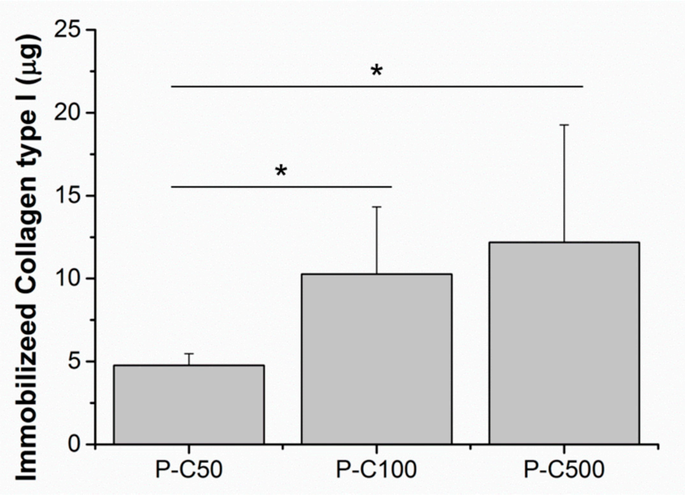

Figure S1. Encapsulated collagen type I within scaffolds immobilized at concentrations of 50 (P-C50), 100 (P-C100), and 500 (P-C500) μg/mL. * Both protein amounts of P-C100 and P-C500 were more than that of P-C50 ($P < 0.05$). Error bar represents standard deviation of means; $n = 3$.

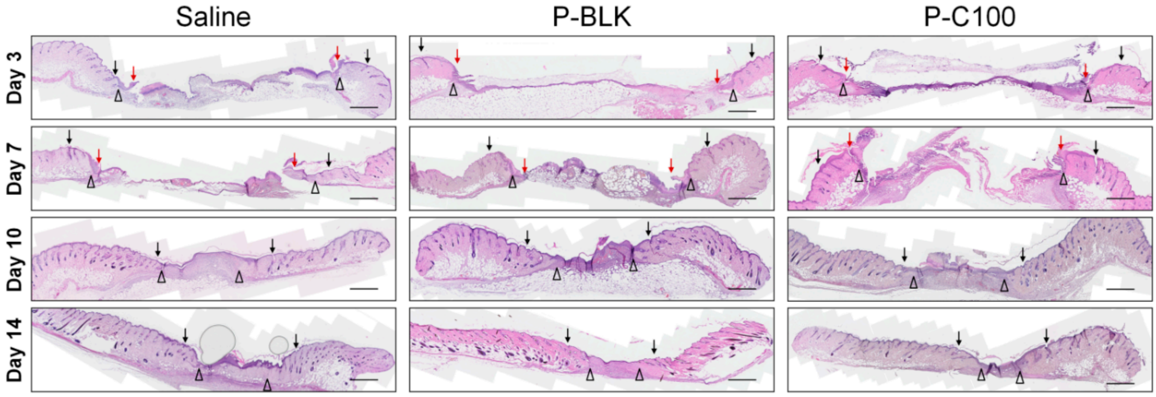

Figure S2. Representative hematoxylin and eosin images of wound biopsy sections from saline treated, P-BLK treated, P-C100 treated mice at indicated days post-wounding. Scale bar = 1000 μm. The black arrows indicate the first hair follicles on the wound edge. The red arrows indicate the advancing margins of neo-epidermis. The triangle symbols indicate the cut ends of skin connective tissue.
